# Developmental onset distinguishes three types of spontaneous recognition memory in mice

**DOI:** 10.1038/s41598-020-67619-w

**Published:** 2020-06-30

**Authors:** Arely Cruz-Sanchez, Shadini Dematagoda, Ridda Ahmed, Sakhithya Mohanathaas, Nicole Odenwald, Maithe Arruda-Carvalho

**Affiliations:** 1 Department of Psychology, University of Toronto Scarborough, Toronto, M1C1A4 Canada; 2 Department of Cell and Systems Biology, University of Toronto Scarborough, Toronto, M1C1A4 Canada

**Keywords:** Neuroscience, Psychology

## Abstract

Spontaneous recognition memory tasks build on an animal’s natural preference for novelty to assess the *what*, *where* and *when* components of episodic memory. Their simplicity, ethological relevance and cross-species adaptability make them extremely useful to study the physiology and pathology of memory. Recognition memory deficits are common in rodent models of neurodevelopmental disorders, and yet very little is known about the expression of spontaneous recognition memory in young rodents. This is exacerbated by the paucity of data on the developmental onset of recognition memory in mice, a major animal model of disease. To address this, we characterized the ontogeny of three types of spontaneous recognition memory in mice: object location, novel object recognition and temporal order recognition. We found that object location is the first to emerge, at postnatal day (P)21. This was followed by novel object recognition (24 h delay), at P25. Temporal order recognition was the last to emerge, at P28. Elucidating the developmental expression of recognition memory in mice is critical to improving our understanding of the ontogeny of episodic memory, and establishes a necessary blueprint to apply these tasks to probe cognitive deficits at clinically relevant time points in animal models of developmental disorders.

## Introduction

The ability to detect the prior occurrence of a given stimulus, or recognition memory, is an intrinsic facet of declarative memory, and is essential to guide future behavior. Behavioral tasks for measuring spontaneous recognition memory are well established^1–4^, easily generalized across species^5,6^, and ethologically relevant, as they explore an animal’s natural preference for novelty. As such, they offer an important foundation for animal models of neurodevelopmental, neurodegenerative and psychiatric disorders^7,8^. While most spontaneous recognition memory studies in rodents use adult animals, recognition memory deficits have been consistently reported in animal models of early onset disorders such as schizophrenia^8–11^ and autism spectrum disorders^12–15^, signaling a need for improved understanding of the developmental regulation of recognition memory in the context of these disorders. The diversity and increasing accessibility of genetic manipulations make the mouse a valuable model for the study of neurodevelopmental disorders, and yet to our knowledge only two papers have examined recognition memory across early development in mice^16,17^.

Three spontaneous recognition memory tasks commonly used to assess rodent models of disease^8,10,18–21^, novel object recognition (NOR), object location (OL), and temporal order recognition (TOR), are used to explore the *what, where* and *when* dimensions of recognition memory. All three tasks involve the spontaneous exploration of object sets in a chamber, with different categories of novelty introduced in each task. In NOR, animals are presented with a novel object (*what*); in OL one of the familiar objects is moved to a novel spatial location (*where*); in TOR, animals are exposed to objects they have interacted with at different points in time (*when*). While similar in their basic elements, these three tasks vary not only in the type of recognition memory they assess, but also in their engagement of different brain regions, including hippocampus, perirhinal and prefrontal cortex^22,23^. Early postnatal life is marked by significant morphological and synaptic development within these brain areas^24–26^, however the impact of this maturation on the ontogeny of recognition memory is unknown. One hypothesis is that the timing of task emergence will follow known maturation trajectories of associated brain regions. Accordingly, tasks relying on brain regions with relatively delayed maturation, such as prefrontal cortex, would similarly display a delay in emergence. To test this hypothesis, we sought to establish the timeline of task-specific ontogeny for distinct forms of spontaneous recognition memory in mice.

Efforts to establish the developmental onset of spontaneous recognition memory tasks in mice^16^ and rats^27–35^ have yielded conflicting results, with differences in species (rat vs. mouse), rat strain and task design (number of objects, prior experience in one or more recognition memory task) likely contributing to the inconsistencies. Thus, we sought to directly compare the ontogeny of three types of spontaneous recognition memory in parallel in C57/129J mice. We found that spontaneous recognition memory tasks differed in their age of onset. Mice were first able to detect changes in spatial location of the objects (OL), followed by distinguishing a novel object (NOR) at a 24 h interval, with recency recognition (TOR) the last to emerge. Our results define distinct temporal signatures for the onset of subtypes of spontaneous recognition memory in mice, pointing to behavior-specific maturational trajectories that may reflect the ontogeny of circuit-behavior relationships.

## Results

**Figure 1:**
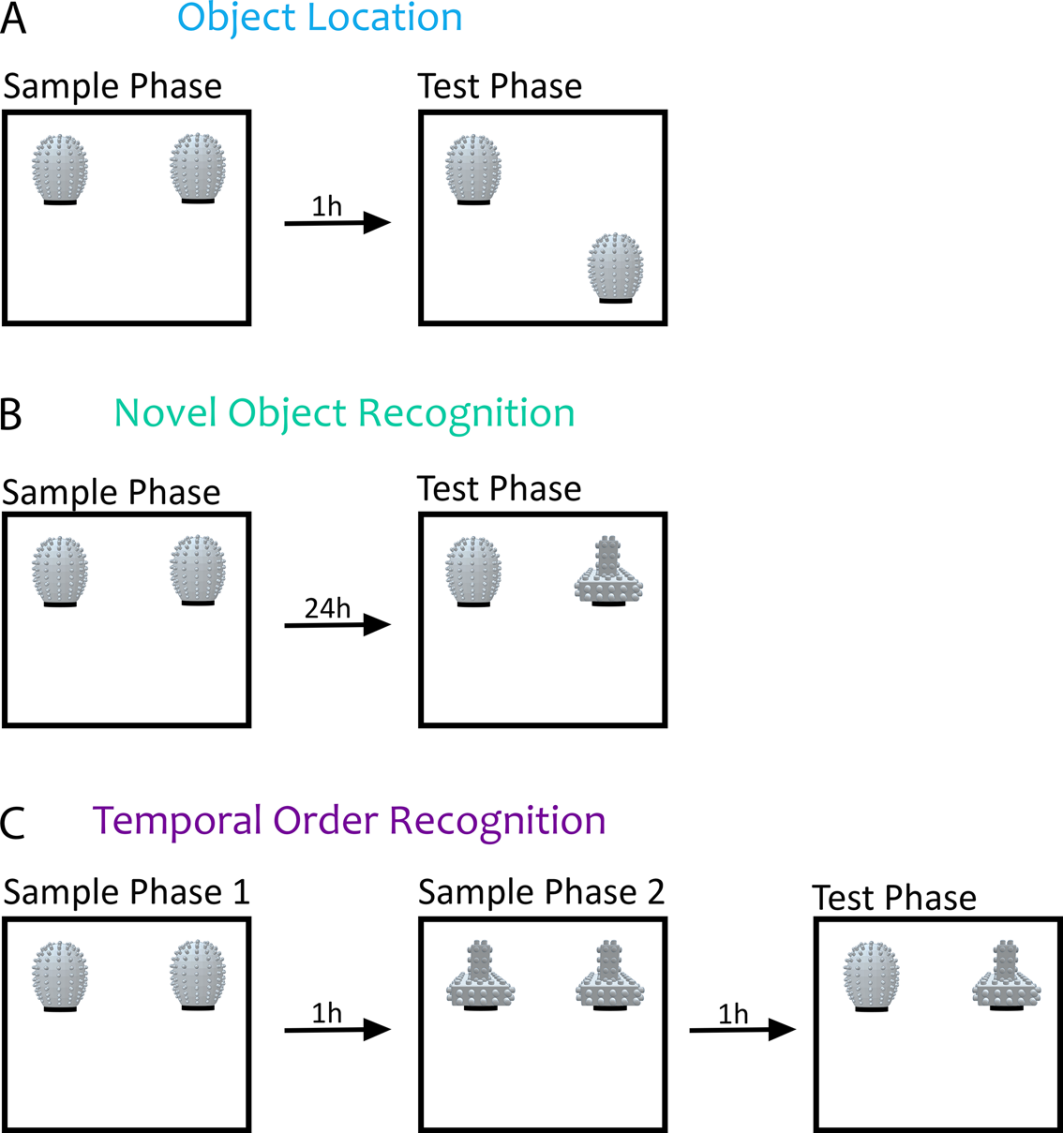
Experimental design for object recognition tasks. (**A**) Schematic diagram of the object location task (OL). Mice of different ages underwent a 10 min sample phase and, following a 1 h delay period, underwent a 5 min test phase in which one object was moved to a novel location. (**B**) Schematic diagram of the novel object recognition task (NOR). Mice of different ages underwent a sample phase that terminated once a criterion of 20 s total object exploration time was met. Following a 24 h delay, mice underwent a 5 min test phase in which one object was replaced with a novel object. (**C**) Schematic diagram of the temporal order recognition task (TOR). Mice of different ages underwent two sample phases with an inter-phase delay of approximately 1 hour. Following another hour delay, mice underwent a 5 min test phase where they interacted with one object presented in sample phase 1 and one object presented in sample phase 2.

To examine the onset of different types of spontaneous recognition memory in mice, we subjected three independent cohorts of mice to OL, NOR, or TOR behavioral tasks. All three tasks consist of a sample or initial exposure phase, followed by a test phase in which novelty is introduced. In the object location (OL) task, animals are first exposed to two copies of the same object in the sample phase (Fig. 1A). In the test phase, one of the objects is moved to a novel spatial location (Fig. 1A). The animal’s ability to detect the change in location is interpreted as more time spent exploring the displaced object. Novel object recognition (NOR) follows a similar sample phase to OL, and in the test phase one of the familiar objects is replaced by a novel object (Fig. 1B). The animal’s ability to recognize the novel object as distinct is interpreted as more time spent exploring the novel compared to the familiar object. In TOR, animals are first exposed to a set of two identical objects in sample phase 1, followed by a novel set of two identical objects in sample phase 2 (Fig. 1C). In a subsequent test phase, animals are exposed to replicas of one object from the sample phase 1 (old) object and one object from the more recent sample phase 2 (Fig. 1C). The underlying assumption of the TOR task is that, provided the animal can distinguish how recently it explored each object, it should show a preference for the object it was less recently exposed to (old object).

To systematically probe the developmental emergence of OL, NOR and TOR, we chose up to 5 time points (postnatal day (P)16, P21, P25, P28 and P35) spanning late infancy to adolescence^36^, starting a few days after eye opening^37,38^. Independent cohorts of animals were tested in OL, NOR or TOR.

### Object location

**Figure 2:**
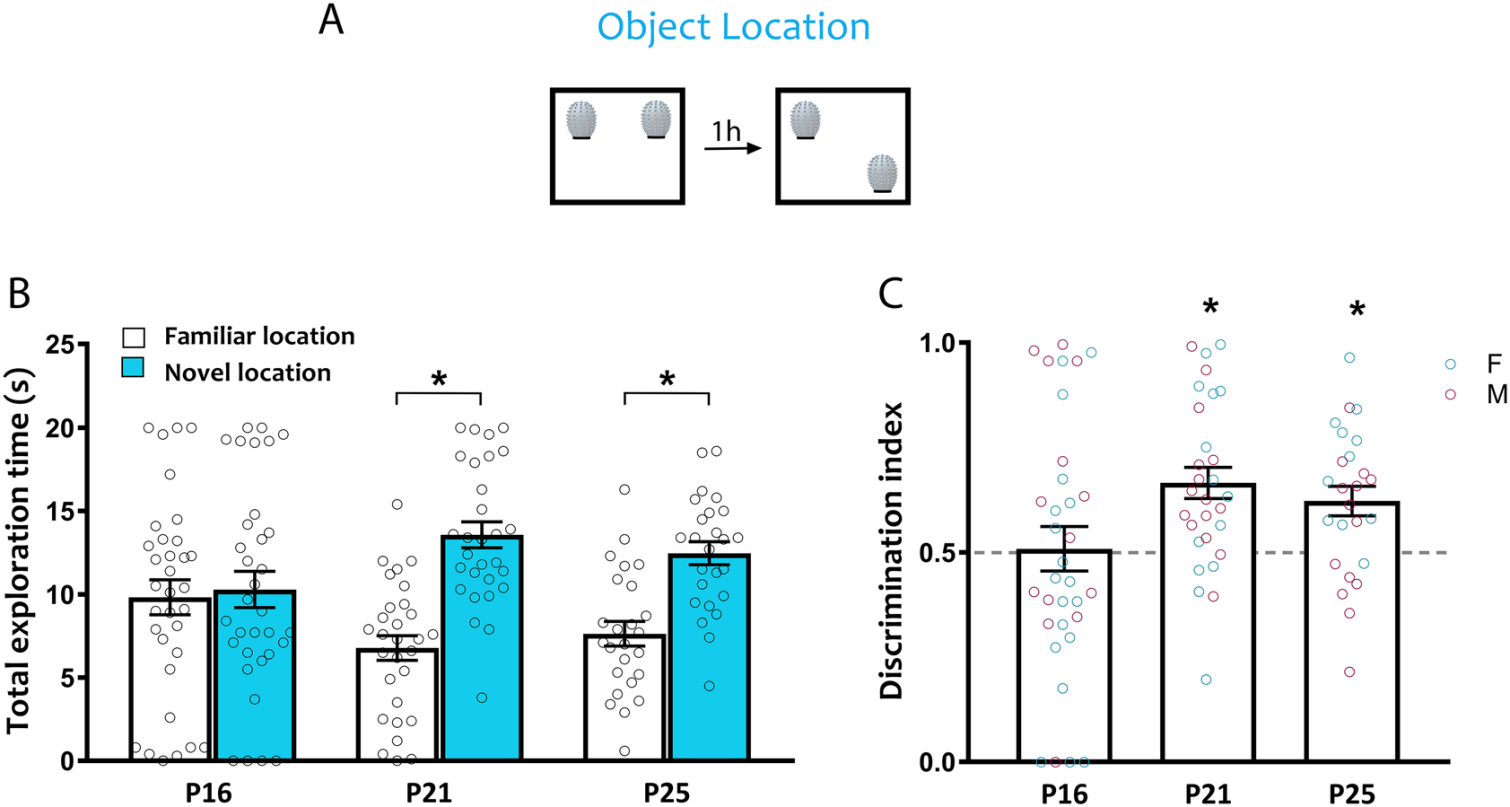
Ontogeny of object location recognition memory. C57/129J mice were tested in the OL task at P16, P21 or P25. (**A**) Schematic of the OL task. (**B**) Object exploration during the test phase of the OL task. Only P21 and P25 mice spent significantly more time exploring the novel location compared to the familiar location. (**C**) Relative preference for the novel location was calculated by a discrimination index (DI) dividing the time spent exploring the new location by the total object exploration time throughout the first 20 s of object interaction. A preference for the novel location was observed only at P21 and P25. Female (cyan) and male (magenta) data points are identified, indicating the lack of observed sex differences. *p < 0.05. P16, *n* = 33 (19 females, 14 males); P21, *n* = 29 (14 females, 15 males); P25, *n* = 25 (12 females, 13 males).

To determine when mice can first recognize a change in the spatial location of an object, we tested P16, P21 and P25 C57/129J mice in the OL recognition task (Fig. 2A). While P16 mice explored the familiar and novel locations similarly, P21 and P25 mice spent more time exploring the object in the novel location (Fig. 2B; two way ANOVA age × location *F*_(2,84)_ = 3.48, *p* = 0.035; P16: *t*_(84)_ = 0.30, *p* = 0.99; P21: *t*_(84)_ = 3.81, *p* = 0.0008; P25: *t*_(84)_ = 2.48, *p* = 0.045). To determine whether mice were individually expressing a preference for the novel over the familiar location, we calculated a discrimination index by dividing the amount of time spent exploring the novel location by the total time spent exploring both objects. This analysis allows for the comparison of relative preference controlling for variability associated with individual differences in exploration. Discrimination indices in P16 mice were close to chance level (0.5), with evidence of discrimination emerging at P21 and still evident at P25 (Fig. 2C; one way ANOVA, *F*_(2,84)_ = 3.56, *p* = 0.033; P16: *t*_(64)_ = 0.17, *p* = 0.87; P21: *t*_(56)_ = 4.46, *p* < 0.0001; P25: *t*_(48)_ = 3.48, *p* = 0.0011). We found no sex differences in OL recognition memory (two way ANOVA, effect of sex: *F*_(1,115)_ = 0.002, *p* = 0.95; age × sex interaction: *F*_(3,115)_ = 2.38, *p* = 0.073). These results suggest that the ability to recognize changes in spatial location in the OL task emerges between P16 and P21 in C57/129J mice.

### Novel object recognition

**Figure 3:**
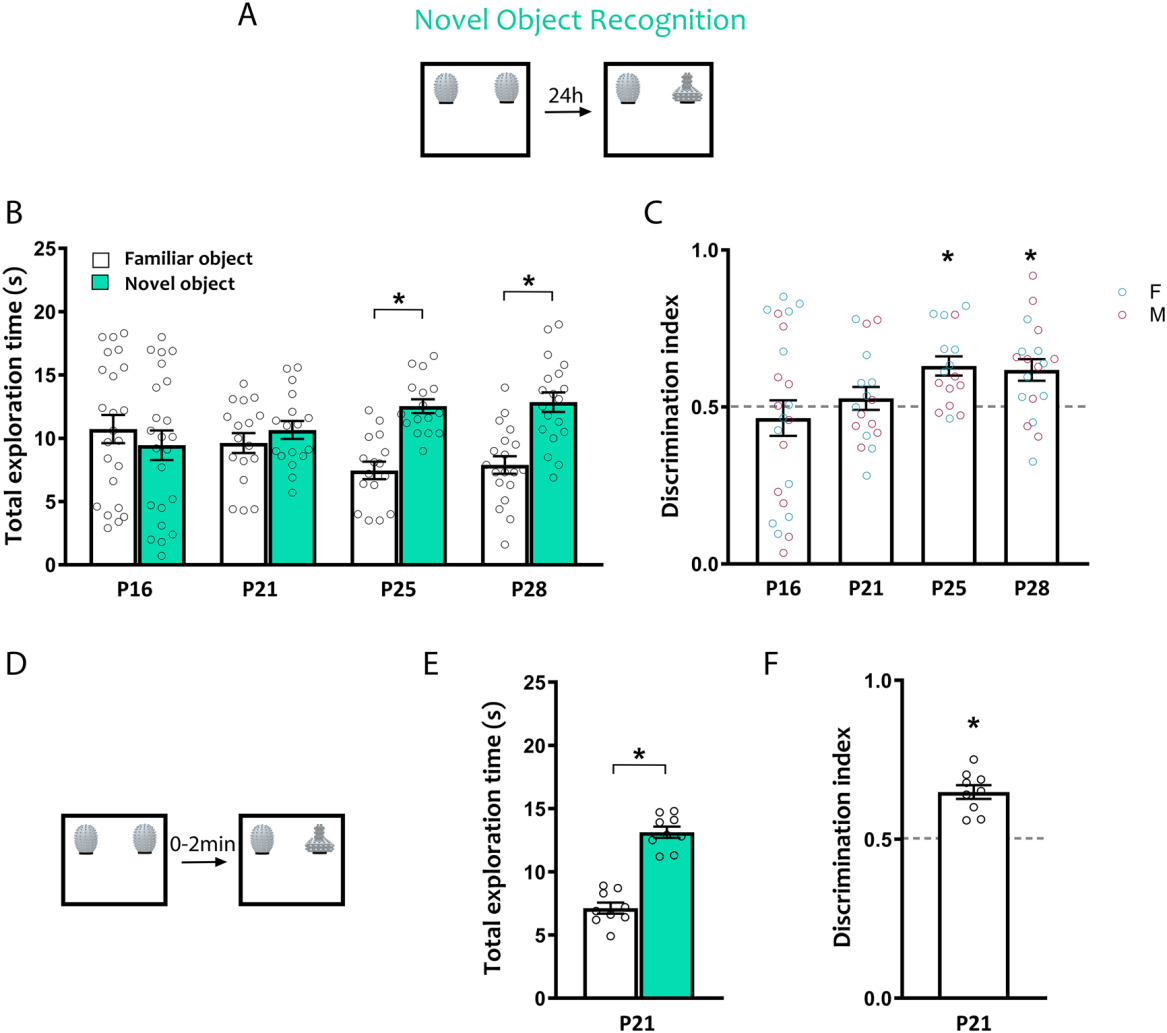
Ontogeny of novel object recognition memory. C57/129J mice were tested in the NOR task at P16, P21, P25 or P28. (**A**) Schematic of the NOR task. (**B**) Object exploration during the test phase of the NOR task. Only P25 and P28 mice spent significantly more time exploring the novel object compared to the familiar object. (**C**) Relative preference for the novel object was calculated as a discrimination index (DI) dividing the time spent exploring the new object by the total object exploration time throughout the first 20 s of object interaction. A preference for the novel object was observed only at P25 and P28. (**D**) Schematic of the NOR task with immediate delay. P21 mice underwent the same NOR protocol except with an immediate delay (under 2 min). (**E**) Object exploration during the test phase of the NOR immediate delay task during the first 20 s of object exploration. (**F**) Relative preference for the novel object during the test phase of the NOR immediate delay task expressed as a DI. P21 animals that underwent an immediate delay displayed preference for the novel object. Female (cyan) and male (magenta) data points are identified, indicating the lack of observed sex differences. *p < 0.05. P16, *n* = 23 (12 females, 11 males); P21, *n* = 17 (9 females, 8 males); P25, *n* = 16 (8 females, 8 males); P28, *n* = 19 (9 females, 10 males). P21 immediate delay, *n* = 9 (2 females, 7 males).

We then asked when the ability to recognize and retain the memory of a novel object in a familiar context for 24 h emerges, testing mice at P16, P21, P25 and P28 in the NOR task (Fig. 3A). P16 and P21 mice explored both familiar and novel objects equally, while P25 and P28 mice spent more time exploring the novel object (Fig. 3B; two-way ANOVA age × object *F*_(3,71)_ = 3.25, *p* = 0.027; P16: *t*_(71)_ = 0.80, *p* = 0.89; P21: *t*_(71)_ = 0.55, *p* = 0.97; P25: *t*_(71)_ = 2.64, *p* = 0.040; P28: *t*_(71)_ = 2.81, *p* = 0.025). Comparison of discrimination indices revealed that P16 and P21 mice did not discriminate above chance levels (0.5), and preference for the novel object was evident at both P25 and P28 (Fig. 3C; one-way ANOVA, *F*_(3,71)_ = 3.31, *p* = 0.025; P16: *t*_(44)_ = 0.62, *p* = 0.54; P21: *t*_(32)_ = 0.75, *p* = 0.46; P25: *t*_(30)_ = 4.26, *p* = 0.0002; P28: *t*_(36)_ = 3.43, *p* = 0.0015). Importantly, when we conducted NOR with a minimum delay (shorter than 2 min), P21 animals displayed a preference for the novel object (Fig. 3D, E: exploration time, paired t test *t*_(8)_ = 6.9, *p* = 0.0001; Fig. 3F: discrimination index, unpaired t test *t*_(18)_ = 7.64, p < 0.0001), indicating that these age-dependent changes in performance are driven by changes in recognition memory, and not sensory or motor abilities necessary to complete the task. We did not observe sex differences in NOR (two way ANOVA, effect of sex: *F*_(1,67)_ = 0.47, *p* = 0.49; age × sex interaction: *F*_(3,67)_ = 0.84, *p* = 0.48). These results suggest the ability to recognize a novel object after a 24 h delay in the NOR task emerges between P21 and P25.

### Temporal order recognition

**Figure 4:**
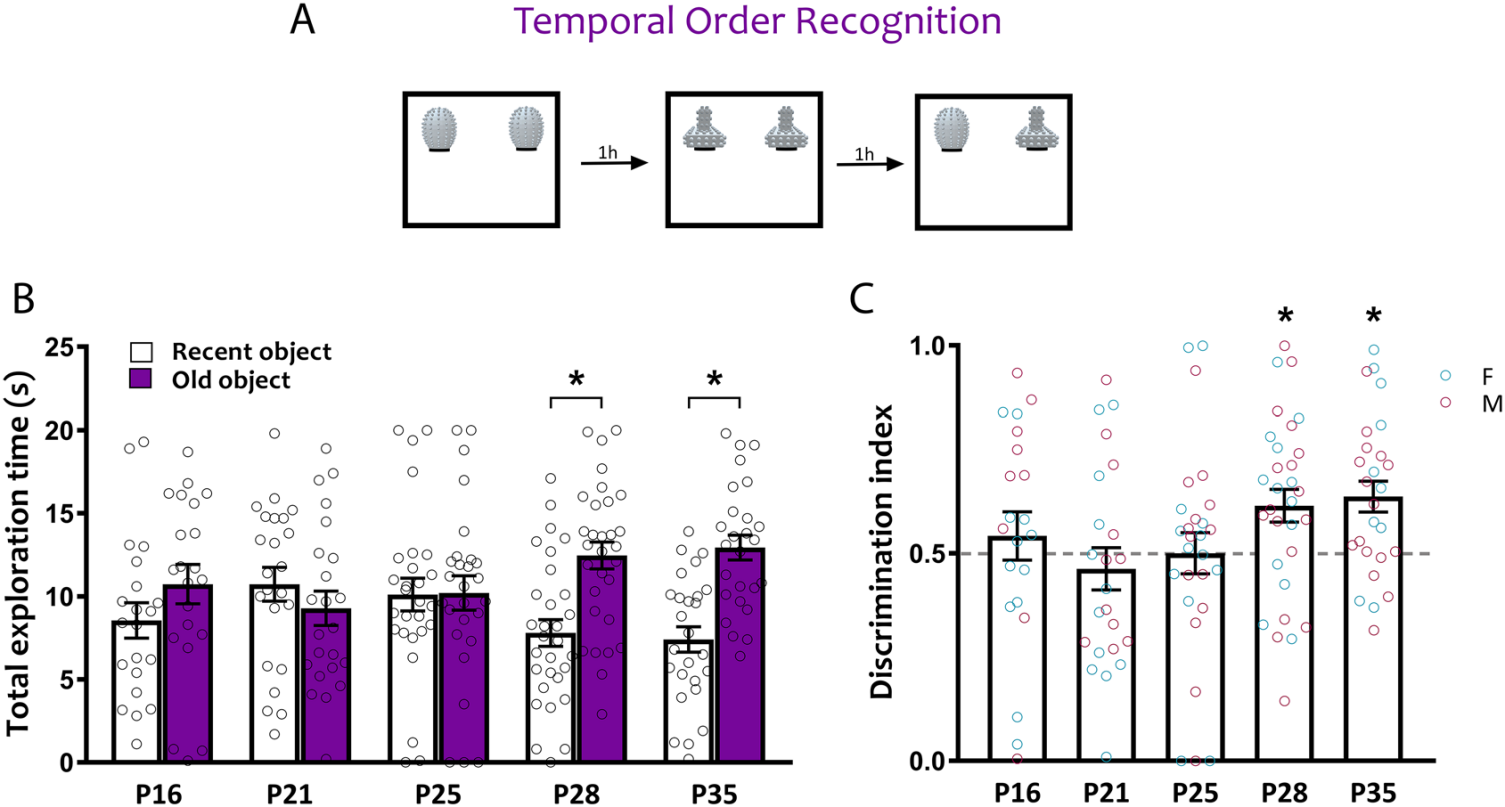
Ontogeny of temporal order recognition memory. C57/129J mice were tested in the TOR task at P16, P21, P25, P28 or P35. (**A**) Schematic of the TOR task. (**B**) Object exploration during the test phase of the TOR task. Only P28 and P35 mice spent significantly more time exploring the old object compared to the recent object. (**C**) Relative preference for the old object was calculated by a discrimination index (DI) dividing the time spent exploring the old object by the total object exploration time throughout the first 20 s of object interaction. A preference for the old object was observed only at P28 and P35. Female (cyan) and male (magenta) data points are identified, indicating the lack of observed sex differences. *p < 0.05. P16, *n* = 21 (12 females, 9 males); P21, *n* = 23 (14 females, 9 males); P25, *n* = 27 (13 females, 14 males); P28, *n* = 30 (13 females, 17 males); P35, *n* = 26 (10 females, 16 males).

To determine when mice form the ability to detect recency among objects explored at different time points, we tested mice at P16, P21, P25, P28 and P35 in the TOR task (Fig. 4A). P16, P21 and P25 mice explored both old and recent objects similarly, whereas P28 and P35 mice spent more time exploring the older object (Fig. 4B; two-way ANOVA age × recency *F*_(4,122)_ = 2.59, *p* = 0.040; P16: *t*_(122)_ = 1.08, *p* = 0.81; P21: *t*_(122)_ = 0.75, *p* = 0.95; P25: *t*_(122)_ = 0.039, *p* > 1; P28: *t*_(122)_ = 2.76, *p* = 0.033; P35: *t*_(122)_ = 3.03, *p* = 0.015). Assessing relative preference by the discrimination index revealed that P16, P21 and P25 mice did not discriminate between the older and recent objects above chance level (0.5) (Fig. 4C; one-way ANOVA, *F*_(4,122)_ = 2.55, *p* = 0.0426; P16: *t*_(40)_ = 0.72, *p* = 0.47; P21: *t*_(44)_ = 0.74, *p* = 0.47; P25: *t*_(52)_ = 0.0042, *p* = 1.0), while P28 and P35 exhibited a preference for the older object (Fig. 4C; P28: *t*_(58)_ = 2.91, *p* = 0.0051; P35: *t*_(50)_ = 3.67, *p* = 0.0006). No sex differences were observed (two way ANOVA, effect of sex*: F*_(1,117)_ = 0.13, *p* = 0.72; age × sex interaction: *F*_(4,117)_ = 0.73, *p* = 0.58). These results suggest the ability to recognize recency in the TOR task emerges between P25 and P28 in C57/129J mice.

### Total exploration

**Figure 5:**
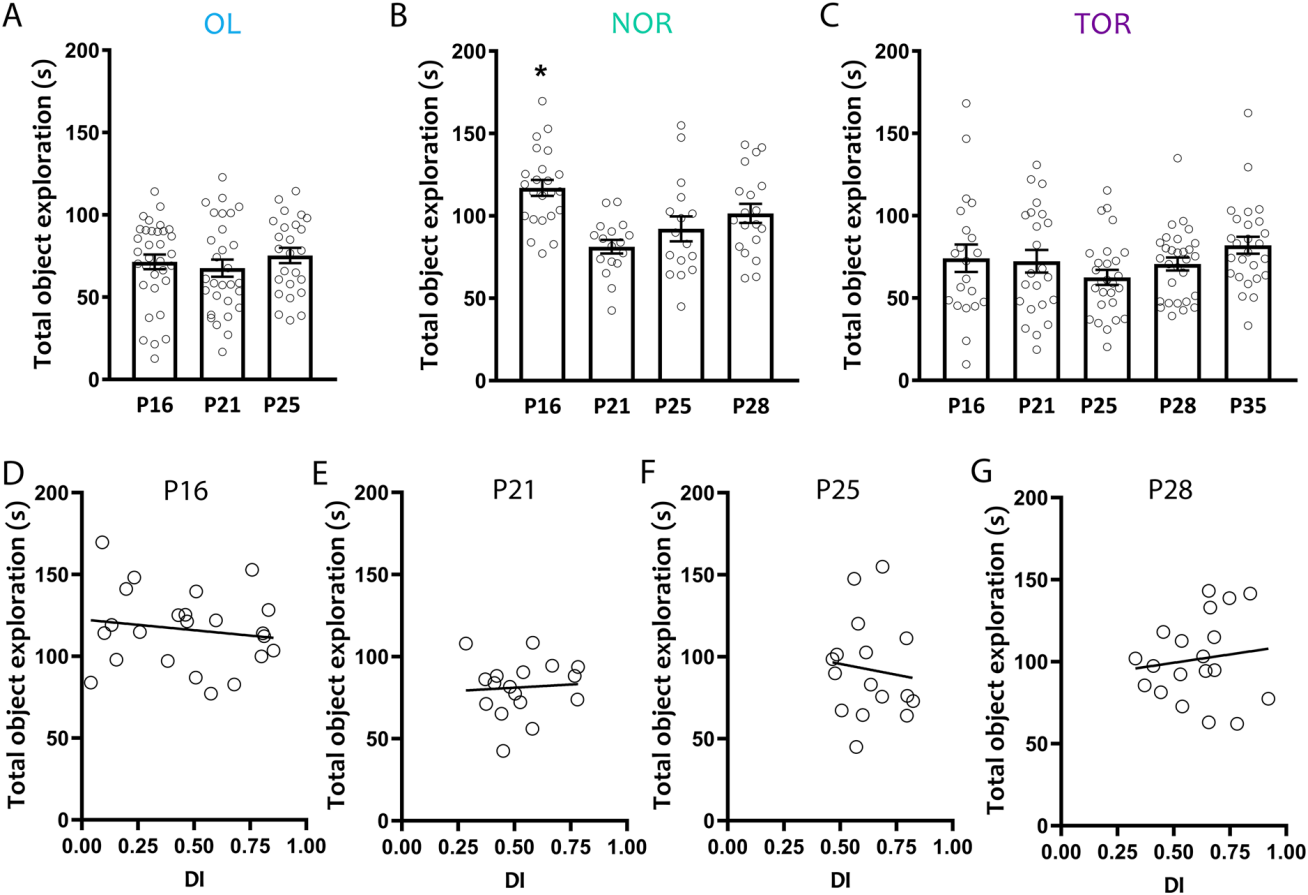
Total object exploration across ages and tasks. Total object exploration during the test phase for (**A**) Object location (OL), (**B**) Novel object recognition (NOR) and (**C**) Temporal order recognition (TOR). We found no differences in object exploration time among any of the age groups for OL and TOR. P16 mice showed increased object exploration compared to P21 and P25 mice in NOR. (**D–G**) Correlation between total object exploration during the 5 min test phase and the discrimination index (DI) for all ages of the NOR task. No correlation was found for any of the age groups suggesting that behavioral performance in NOR is not influenced by differences in object exploration. *p < 0.05. OL: P16, *n* = 33; P21, *n* = 29; P25, *n* = 25; NOR: P16, *n* = 23; P21, *n* = 17; P25, *n* = 16; P28, *n* = 19; TOR: P16, *n* = 21; P21, *n* = 23; P25, *n* = 27; P28, *n* = 30; P35, *n* = 26.

To further test whether age-dependent changes in discrimination were specific to recognition memory, and not driven by changes in other task components such as motivation, we analyzed the object exploration times in all three tasks in the full 5 min of the test phase. Mice spent the same amount of time engaging in object exploration in all sampled ages in the OL (Fig. 5A; one way ANOVA, *F*_(2,84)_ = 0.58, *p* = 0.56) and TOR (Fig. 5C; one way ANOVA, *F*_(4,122)_ = 1.64, *p* = 0.17), suggesting age-dependent changes in discrimination in these tasks are not due to lack of motivation or other factors affecting object exploration. Surprisingly, P16 mice spent more time exploring objects in the NOR task compared to all other ages (Fig. 5B; one way ANOVA, *F*_(3,71)_ = 7.68, *p* = 0.0002; P16 vs P21, *p* = 0.0001; P16 vs P25, *p* = 0.013). To exclude the possibility that developmental changes in the time spent exploring the objects could underlie the changes in novel object preference in NOR, we probed the relationship between total exploration time and discrimination index (Fig. 5D–G). We found no significant correlation between total object exploration and performance in NOR in P16 mice (Fig. 5D; r = − 0.15, *p* = 0.49), suggesting increased exploration is not driving impaired discrimination in this age group. Total exploration time and performance were similarly not significantly correlated in the remaining ages in NOR (Fig. 5E; P21, r = 0.069, *p* = 0.79; Fig. 5F; P25, r = − 0.11, *p* = 0.69; Fig. 5G; P28, r = 0.13, *p* = 0.60), OL (P16, r = − 0.060, *p* = 0.74; P21, r = 0.32, *p* = 0.092; P25, r = − 0.19, *p* = 0.37) or TOR (P16, r = 0.095, *p* = 0.68; P21, r = 0.18, *p* = 0.42; P25, r = − 0.22, *p* = 0.28; P28, r = 0.041, *p* = 0.83; P35, r = − 0.11, *p* = 0.60).

Similarly, we saw no age-dependent differences in sample phase object exploration times between ages in OL (Supplementary Fig. 1A, one-way ANOVA, *F*_(2,84)_ = 2.36, *p* = 0.10; Supplementary Fig. 1B two-way ANOVA age × location *F*_(2,84)_ = 2.83, *p* = 0.064) or TOR, (Supplementary Fig. 3A, B, sample phase 1: one-way ANOVA, *F*_(4,122)_ = 2.56, *p* = 0.06; sample phase 2: one-way ANOVA, *F*_(4,122)_ = 0.97, *p* = 0.42; Supplementary Fig. 3C, D, sample phase 1: two-way ANOVA age × object *F*_(4,122)_ = 2.136, *p* = 0.080; sample phase 2: two-way ANOVA age × object *F*_(4,122)_ = 0.21, *p* = 0.93), time to criterion in sample phase in NOR (Supplementary Fig. 2A, one-way ANOVA, *F*_(3,71)_ = 1.62, *p* = 0.19; Supplementary Fig. 2B, two-way ANOVA age × object *F*_(3,71)_ = 1.61, *p* = 0.19), or any correlation between sample phase exploration time and performance (OL Supplementary Fig. 1C–E, P16, r = 0.094, *p* = 0.60; P21, r = 0.31, *p* = 0.10; P25, r = − 0.18, *p* = 0.39; NOR Supplementary Fig. 2C–F, P16, r = − 0.076, *p* = 0.73; P21, r = − 0.47, *p* = 0.054; P25, r = 0.027, *p* = 0.92; P28, r = 0.12, *p* = 0.63; TOR Supplementary Fig. 3E–N, sample phase 1: P16, r = 0.13, *p* = 0.57; P21, r = 0.38, *p* = 0.072; P25, r = 0.015, *p* = 0.94; P28, r = − 0.15, *p* = 0.44; P35, r = − 0.28, *p* = 0.16; sample phase 2: P16, r = 0.087, *p* = 0.71; P21, r = 0.22, *p* = 0.31; P25, r = 0.16, *p* = 0.44; P28, r = − 0.15, *p* = 0.44; P35, r = − 0.13, *p* = 0.53). Overall, these results suggest that any differences in object exploration time likely do not explain better performance in OL, NOR or TOR in the sampled ages.

## Discussion

We conducted a parallel analysis of the ontogeny of three types of recognition memory in the same mouse strain, with equivalent analysis parameters to effectively compare the relative timing of onset for each of these tasks irrespective of variations in species, strain, or animal facility. Applying this systematic approach, we established that C57/129J mice display differential developmental emergence for distinct forms of spontaneous recognition memory. The ability to recognize changes in spatial location (OL) (1 h interval) emerges first, at P21, followed by the ability to retain the memory of distinct object features (NOR) for 24 h at P25, and recognition of the recency of events (TOR, 1 h interval) is the last to emerge, at P28. These data identify precise temporal windows for the onset of differential aspects of spontaneous recognition memory in mice, an essential first step towards examining the neural correlates underlying this developmental sequence.

Most rodent spontaneous recognition memory studies have used adult animals, with a few focusing on adolescence or juvenility^30,33,39–41^. Studies examining spontaneous recognition memory in young rats show OL memory onset at P16^27^ or P17^28^–P21^29–31^ (depending on rat strain, but see^32^), and NOR onset between P23 and P29^33^ for long retention intervals (24 h) as used here, and at P15^27,34^–P18^29,31,35^ at short retention intervals (up to 10 min). Given the difference in memory load between short and long (24 h) intertrial intervals for NOR, it is not surprising that the age of onset differs between these task variants. The earliest reports of TOR in the rat are at P17^27^ and P20^31^. These are largely consistent with our results in showing earlier onset of OL relative to NOR (at a 24 h retention interval), but suggest earlier onset of TOR in rats compared to mice (P17–20 compared to P28 in our study). Importantly, we cannot exclude a role for differences in experimental design in this discrepancy. Specifically, while in our study each task was assessed independently, in both TOR rat studies the same animals had previously undergone NOR and OL^27,31^, leading to different levels of habituation between tasks and the possibility of memory interference. Additionally, both studies had a shorter delay between TOR sessions^27,31^. One study looking at the ontogeny of recognition memory in CD1 mice reported onset of NOR between P18 and P28^16^, but did not examine OL or TOR. Although this suggests a consistent timeline for onset of NOR in both CD1^16^ and C57/129J mouse strains, it is important to note that their study significantly differed from ours in experimental design, featuring a shorter retention interval (2 min), double the number of objects in the arena (four) and prior experience in an object-place manipulation^16^. Consistent with our findings for OL, Bath and colleagues reported OL memory (25 min delay) at P21 in male C57Bl/6N mice, but saw a delayed onset in females^17^.

We found no age-dependent changes in total object exploration time with the exception of an increase in exploration time in P16 mice in NOR. One difference of NOR compared to the other tasks is the shorter sample phase object exploration time (20 s). It is possible that reduced opportunity for exploration at the sample phase contributed to an increase in exploration in the test phase. Indeed, overall test phase exploration time is slightly higher in NOR compared to other tasks. However, it is unclear why this would differentially affect P16 animals. This result was particularly surprising given prior work in rats^29^ and CD1 mice^16^ describing reduced exploration in preweaning animals. This inconsistency could be due to differences in handling and habituation (one^16^ to three^29^ sessions in previous studies, compared to our eight sessions over 4 days), and/or species and mouse strain, with the latter being known to significantly affect exploration time in spontaneous tasks in adult mice^42^. The lack of correlation between total exploration and discrimination index suggests that exploration is not a primary factor limiting performance in young C57/129J mice. Consistent with previous studies^16,41,43^, we also did not observe sex differences in any of our tasks. To our knowledge, sex differences in recognition memory in mice^44^ and rats^45^ have mostly been reported in animals at older ages, suggesting male and female mice may perform equally in spontaneous object recognition tests within the juvenile period. One exception is the study by Bath and colleagues that sees a delay in the onset of OL in female C57BL/6N mice^17^.

What may underlie the differential onset of each of these forms of recognition memory? The lack of a correlation between total object exploration and discrimination suggests that, in C57/129J mice, preferences emerge as a result of recognition of specific stimulus features. Lesion and pharmacological studies point to circuit specialization for the memory processes probed in each of our three recognition memory tasks^22^, with primary involvement of hippocampus^46–49^ in OL, perirhinal cortex^49–54^ in NOR and connections^48,53–56^ between hippocampus^47,48,57–59^, perirhinal^53,54^ and prefrontal cortex^53,60^ in TOR. One possibility is that brain region-specific maturation dictates the onset of each of these behavioral competencies. This would imply that the observed asynchrony in the onset of each behavior is mediated by differential timing of circuit maturation.

Little is known about the functional maturation of brain circuits underlying recognition memory. In terms of spatial navigation necessary for OL memory, rat head direction and place cell systems feature adult-like patterns as early as P17, with grid cells following at P21^26,61,62^, paralleling the emergence of OL. While this level of spatial representation may be sufficient to sustain OL memory, the number of cells displaying adult-like firing continues to increase through postnatal weeks 4–5^26,61,62^, perhaps accounting for the later emergence of more complex tasks such as object-place^16,61^, object-place-context^63^ and the use of distal visual cues^64–66^. Perirhinal cortex anatomical development, although not extensively studied, is comparable to other neocortical regions^24^. Interestingly, there is evidence for perirhinal requirement for NOR being delay-dependent, with lesions only impairing performance for delays of 10 min or more^51,52^, suggesting the earlier emergence of NOR memory for short delays^27,29,31,34,35^ may be perirhinal-independent. It is important to note that this 24 h interval for NOR differs from the 1 h intertrial interval used in the other tasks in this study, and may differ from the age of onset for 1 h NOR. Prefrontal cortex develops later than other cortical structures^25^, with cytoarchitectonic development reaching adult laminar appearance by P18 in the rat^67^, and volumetric changes stabilizing at P30^68^. Similarly, prefrontal network activity emerges later than in sensory areas, with marked changes in hippocampus-prefrontal activity within the first postnatal weeks^69^. It is tempting to speculate that the delayed onset of TOR reflects the delayed maturation of prefrontal cortex relative to other brain structures.

These data define the developmental emergence of three types of spontaneous recognition memory in C57/129J mice, a tool broadly useful for the interrogation of memory function during early life and its implications in neurodevelopmental disorders. The distinct temporal profile of each task further underlines the notion of memory as multifactorial, and recognition memory encompassing several underlying processes rather than being unitary. Future work delineating the anatomical and synaptic maturation of the brain regions underlying different types of spontaneous recognition memory will be key to establishing how circuit-behavior relationships emerge in development, and how they may shape behavior across the lifespan.

## Methods

### Animals

Mice were a cross between C57BLK/6J (maternal) × 129S1/SvImJ (paternal) strains (Jackson Laboratory; referred to as C57/129J for simplicity). Mice were bred at the University of Toronto Scarborough and kept on a 12 h light/dark cycle (lights on at 07:00 h) with access to food and water ad libitum. Date of birth was designated postnatal day (P)0, with litter sizes ranging from 2 to 11 pups. All litters were randomly divided and evenly distributed across ages and by sex, with a minimum of 2 ages/litter and a maximum of 4 littermates/age (in very large litters) to limit potential litter effects. Mice were assigned to 1 of 5 possible age groups depending on the recognition memory task: P16, P21, P25, P28 or P35. At 21 days (P21), mice were weaned and housed in same-sex littermate groups of 2–5 mice. A previous study by Westbrook and colleagues established that weaning does not affect recognition in OL or NOR^29^. All experiments were conducted during the light cycle. Approximately equal numbers of females and males were used for each age group. All animal procedures were approved by the Animal Care Committee at the University of Toronto.

### Apparatus and objects

All recognition memory tests were conducted in a 30 × 30 × 30 cm white plexiglass square chamber with a magnetic, glossy, removable base. The base had a 30 × 30 cm black grid composed of 1 × 1 cm squares to allow for accurate object placement. The chamber was elevated 41 cm off the floor and a camera was mounted 75 cm above the chamber using a wall mount rack. Objects were designed using SolidWorks and 3D printed using a LulzBot TAZ 6 3D printer with natural PLA filament. A round magnet (35 mm diameter) was glued to the base of the objects to allow for stable attachment to the chamber floor. Both objects had a pegged-surface and consisted of the following dimensions: 46 × 46 × 48 mm (step object), and 47 mm diameter × 48 mm height (dome object) (Fig. 1). Object designs were extensively piloted to generate objects that were (1) equal in surface area, (2) made of the same materials, and (3) for which the animals displayed no innate preference. Object types were counterbalanced for all tasks. There was no bias in exploration time related to object type in the test phase for NOR (dome vs step effect; two-way ANOVA, *F*_(1,67)_ = 0.03, *p* = 0.85) or TOR (dome vs step effect; two-way ANOVA, *F*_(1,122)_ = 0.0013, *p* = 0.97).

### Behavioral testing

#### Handling and habituation

Mice were handled and habituated to the behavioral chamber twice a day for four consecutive days prior to the day of testing for all three recognition memory tasks. Handling took place in the testing room with a minimum 3 h interval between handling sessions. Handling and habituation consisted of 5 min of handling followed by placement into the behavioral chamber for 4 min. A 4 × 4 cm weigh boat with kitten milk replacement (Pet*A*g) was placed at the center of the behavioral chamber during habituation to allow for better acclimation to the chamber. All mice were ear-notched at P12 for identification purposes.

#### General procedures

Male and female C57/129J mice underwent behavioral testing at either P16, P21, P25, P28 or P35, depending on the recognition task. To avoid confounds of repeated testing, dedicated cohorts of mice were used per age and per recognition task, such that each animal was only tested at one age and in one recognition memory task. Behavioral chambers were cleaned with water between phases and subjects, and with 70% ethanol at the end of the day. All mice were kept in the home cage with their parents (preweaning ages) and/or littermates (postweaning) during the 24-h delay period for the NOR task. Pre- and post-weaning mice remained in a separate transport cage during the 1-h delay period for the OL and TOR tasks. For all tasks and phases, mice were placed into the chamber with their head facing the wall located opposite the object location. For the sample phases of all three tasks, as well as for the test phases for NOR and TOR, objects were placed in the northwest and northeast corners of the chamber, 3 cm away from each wall. Object type and side of novel stimulus (i.e. novelty in the form of novel location, novel object or old vs recent object was introduced in the right or left side of the cage) were counterbalanced. To further validate lack of a side/location bias, we confirmed that animals did not display a side preference in the test phase in OL (two-way ANOVA, *F*_(1,81)_ = 1.25, *p* = 0.27), NOR (two-way ANOVA side effect, *F*_(1,67)_ = 0.35, *p* = 0.55), or TOR (two-way ANOVA side effect, *F*_(1,117)_ = 0.25, *p* = 0.62).

#### Specific procedures

##### Object location (OL) task

OL was divided into one sample phase followed by a test phase (Fig. 1A). In the 10-min sample phase, mice interacted with two copies of an identical object, after which animals were removed and placed back into their transport cage. After a 1 h delay period^70,71^, mice underwent a 5 min test phase in which they were placed in the chamber with the same two objects, but with one relocated to a novel location (Fig. 1A). The novel location was at the opposite corner of the previous location (south corner, counterbalanced for side), 3 cm away from each wall (Fig. 1A).

##### Novel object recognition (NOR) task

NOR was divided into one sample phase followed by a test phase. The sample phase consisted of placing the mouse into the chamber containing two copies of a single object (Fig. 1B). The sample phase lasted until a criterion of total object exploration of 20 s was reached^72^, at which point the mouse was removed and placed back into the home cage. Following a delay period of either 24 h^72–74^ or an immediate delay (lasting less than 2 min), mice underwent a 5 min test phase where they were placed in a chamber containing both the previously encountered object and a novel object. Mice were returned to the home cage in between all phases of the experiment. We chose a longer delay (24 h) for this task because the brain circuits underlying NOR with shorter delays are not as well characterized^51,52^. This 24 h delay, albeit different from the delay used in OL and TOR, features robust perirhinal involvement even in instances of highly dissimilar objects^52^. Since the present dataset cannot determine whether our objects’ level of feature ambiguity recruits perirhinal cortex at shorter delays, using a 24 h interval should overcome that limitation. Objects for both sample and test phases were positioned as described above under general procedures. Total object exploration measurements took into account the complete test phase, lasting 5 min.

##### Temporal order recognition (TOR) task

TOR was divided into two sample phases followed by a test phase (Fig. 1C). Sample phase 1 consisted of exposure to a set of two identical objects for 10 min in the behavioral chamber. Following approximately a 1 h inter-phase interval^49,75,76^, mice underwent sample phase 2 which consisted of 10 min in a chamber containing a second distinct set of two identical objects (Fig. 1C). After another 50 min to 1 h delay period, mice underwent a 5 min test phase in which they were exposed to one copy of the object from sample phase 1 (old object) and one copy of the object from sample phase 2 (recent object) (Fig. 1C). Mice remained in a separate transport cage in between all phases of the experiment. Order of object type (i.e. which object was assigned as old vs recent) was counterbalanced. Objects for both sample and test phases were positioned as described under general procedures.

#### Behavioral analysis

Behavior was analyzed using ANY-maze software. Exploratory activity was defined as in Ref.^72^. Briefly, this was defined as an object-directed gaze while actively sniffing and/or pawing within 2 cm of the object. Sitting on top of the object while sniffing the surrounding air or chewing the object were not considered exploration. All automated scoring was extensively validated through hand-scoring by an experimenter blind to experimental conditions. A discrimination index was calculated as a measure of relative novelty preference by dividing the amount of time spent exploring the novel location/novel object/older object by the total time spent exploring both objects. Leger and colleagues^72^ recommend also using the 20 s criterion of exploration time for the test phase (adapted from^77^). In our pilot experiments, we confirmed this design yielded more consistent results in NOR for C57/129J mice. To allow for a direct comparison between our three tasks, we applied the same criterion to the test phase of OL and TOR. The analysis of the test phase of all three tasks comprised the first 20 s of total interaction time with the objects. This is further supported by studies showing rodents demonstrate a higher preference for the novel object within the first 60–120 s of the test phase^71,78–81^, which corresponds to when mice reached criterion in our sample.

### Statistical analysis

Data are presented as mean ± SEM. All statistical analyses were performed in Graphpad Prism version 8. Potential sex differences, object bias or side preferences were first assessed using a two-way, repeated measures ANOVA and in the absence of effects, data were collapsed across these variables for subsequent analyses. Object exploration time was analyzed by two-way, repeated-measures ANOVA followed by Sidak’s post-hoc tests to compare object exploration within each age group. Potential group differences in discrimination index (DI) were analyzed by one-way ANOVA followed by unpaired t tests comparing DI to chance exploration level of 0.5 as in Ref.^3,78,82^. Total object exploration was analyzed using a one-way ANOVA followed by the software default of Tukey’s post-hoc tests for comparisons between age groups. Pearson’s correlation coefficients were calculated to probe the relationship between variables using linear regression. Since 2-way ANOVA revealed no sex differences in any of our recognition tasks (OL: two way ANOVA, *F*_3,115_ = 2.38, *p* = 0.07; NOR: two way ANOVA, *F*_3,67_ = 0.84, *p* = 0.48; TOR: two way ANOVA, *F*_4,117_ = 0.73, *p* = 0.58), male and female mice data were pooled and analyzed together for all figures. For all analyses, *p* < 0.05 was considered significant.

### Consent for publication

All experiments were performed in accordance with relevant guidelines and regulations from the University of Toronto.

## Supplementary information

Supplementary figure legends

Supplementary figure S1

Supplementary figure S2

Supplementary figure S3
