# Supplementary material for: Developmental onset distinguishes three types of spontaneous recognition memory in mice
Source: Sci Rep. 2020 Jun 30;10:10612. doi: 10.1038/s41598-020-67619-w (PMC7326931; doi:10.1038/s41598-020-67619-w)
Supplement: Supplementary file 1 — Supplementary figure legends [file 41598_2020_67619_MOESM1_ESM.pdf]

Developmental Onset Distinguishes Three Types of Spontaneous Recognition Memory in Mice

Arely Cruz-Sanchez[1,2], Shadini Dematagoda[1], Ridda Ahmed[1], Sakhithya Mohanathaas[1], Nicole Odenwald[1] and Maithe Arruda-Carvalho[1,2*].

[1] University of Toronto Scarborough, Department of Psychology, Toronto, M1C1A4, Canada
[2] University of Toronto Scarborough, Department of Cell and Systems Biology, Toronto, M1C1A4, Canada
[*] m.arrudacarvalho@utoronto.ca

Supplementary materials

Supplementary Figure legends:

Supplementary Figure 1. Sample phase object exploration in the object location recognition task. ***A.*** Total object exploration during the sample phase of the OL task distributed by age group. ***B.*** Sample phase object exploration distributed by left (white bars) and right (blue bars) object per age group. We found no significant differences in total object exploration nor exploration time between left and right objects among any of the age groups. ***C-E.*** Correlation between total object exploration during the 10-minute sample phase and the discrimination index (DI) for all ages in the OL task. P16, *n* = 33; P21, *n* = 29; P25, *n* = 25.

Supplementary Figure 2. Sample phase object exploration and latency to criterion in the novel object recognition task. *A.* Latency to achieve 20 seconds of total object exploration during the sample phase of the NOR task distributed by age group. ***B.*** Sample phase object exploration distributed by left (white bars) and right (green bars) object per age group. We found no significant differences in latency to reach the 20-second criterion nor exploration time between left and right objects among any of the age groups. ***C-F.*** Correlation between latency to criterion during the sample phase and the discrimination index (DI) for all ages of the NOR task. P16, *n* = 23; P21, *n* = 17; P25, *n* = 16; P28, *n* = 19.

Supplementary Figure 3. Sample phase object exploration in the temporal order recognition task. **A, B.** Total object exploration during sample phase 1(***A***) and sample phase 2 (***B***) of the TOR task distributed by age group. **C, D.** Object exploration distributed by left (white bars) and right (purple bars) object per age group for sample phase 1 (**C**) and sample phase 2 (**D**). We found no significant differences in total object exploration nor exploration time between left and right objects among any of the age groups in sample phase 1 or sample phase 2. **E-N**. Correlation between total object exploration during

the 10 minute sample phase and the discrimination index (DI) for all ages of the TOR task for sample phase 1 (***E-I***) and sample phase 2 (***J-N***). P16, *n* = 21; P21, *n* = 23; P25, *n* = 27; P28, *n* = 30; P35, *n* = 26.
