# Supplementary figures and images for: Developmental onset distinguishes three types of spontaneous recognition memory in mice
Source: Sci Rep. 2020 Jun 30;10:10612. doi: 10.1038/s41598-020-67619-w (PMC7326931; doi:10.1038/s41598-020-67619-w)

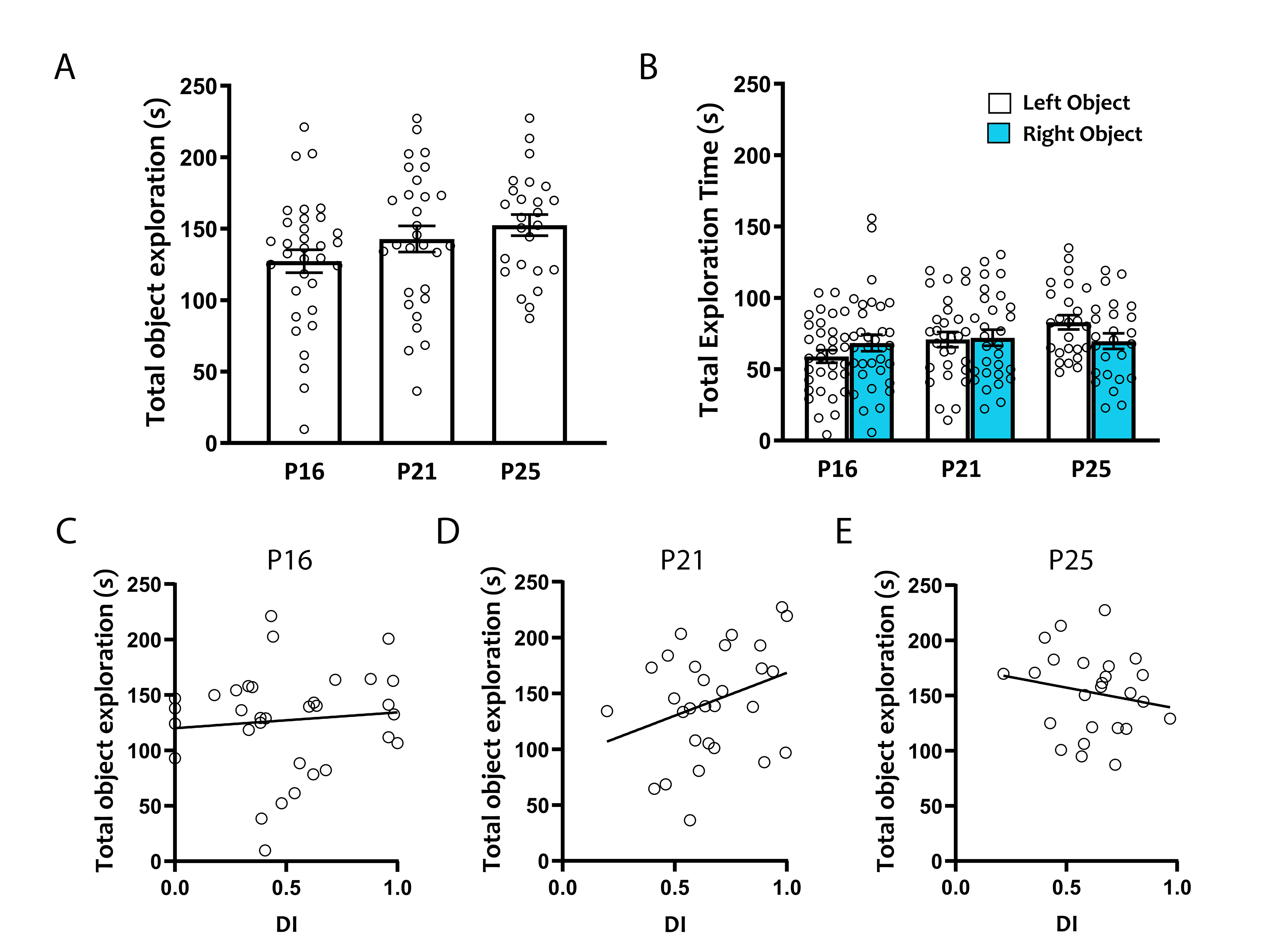

Supplement: Supplementary file 2 — Supplementary figure S1 [file 41598_2020_67619_MOESM2_ESM.tif]

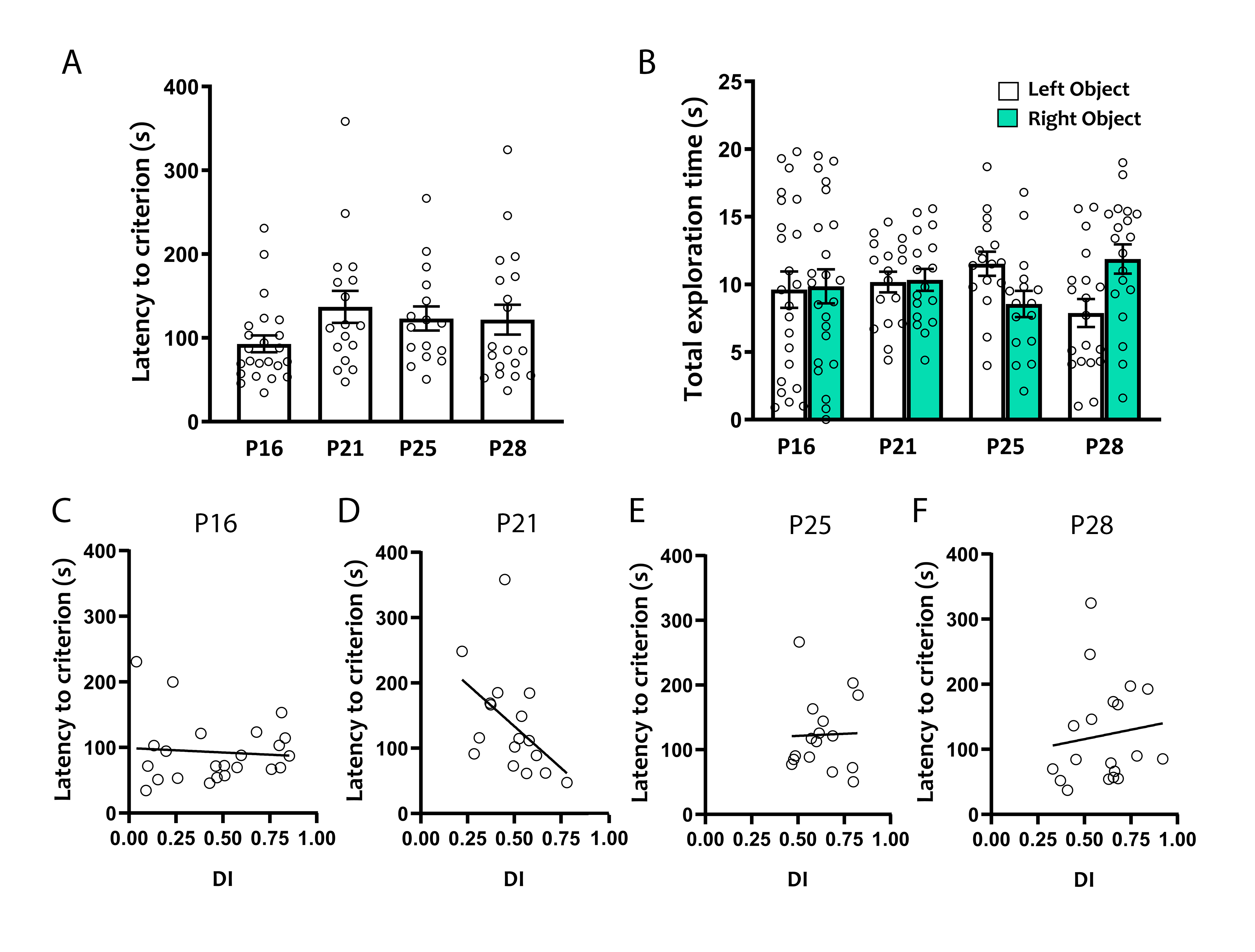

Supplement: Supplementary file 3 — Supplementary figure S2 [file 41598_2020_67619_MOESM3_ESM.tif]

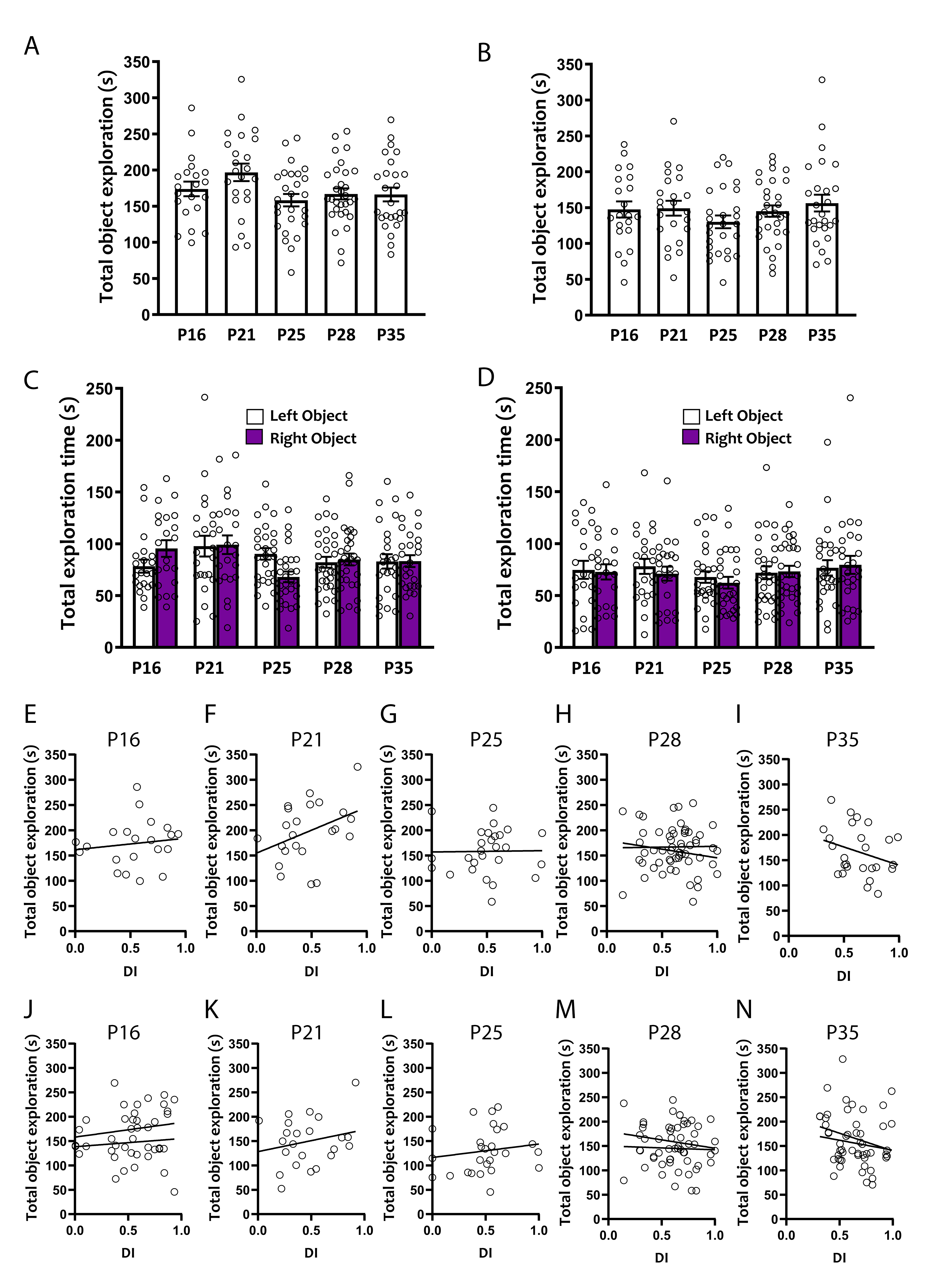

Supplement: Supplementary file 4 — Supplementary figure S3 [file 41598_2020_67619_MOESM4_ESM.tif]
